# Integrated extrusion‐enzymatic treatment of corn bran for production of functional cake

**DOI:** 10.1002/fsn3.738

**Published:** 2018-08-22

**Authors:** Soroush Haghighi‐Manesh, Mohammad Hossein Azizi

**Affiliations:** ^1^ Department of Food Science and Technology School of Agriculture Tarbiat Modares University Tehran Iran

**Keywords:** corn bran, enzymatic treatment, extrusion treatment, functional cake, soluble fiber

## Abstract

Corn bran, as one of the high‐fructose corn syrup industries’ by‐products, is a rich source of functional fibers. The major fraction of corn bran is insoluble arabinoxylan having lower functional properties than the minor soluble function. Therefore, the main aim of this research was to increase the soluble fiber content of corn bran and use it for functional cake production. In this regard, milled corn bran with three different sizes was exposed to one factor enzymatic treatment to select the best sieving size (the criterion for selecting the best product in each stage of assay was production of the highest amount of soluble fiber). Then, milled corn bran with the best particle size was exposed to nine different enzymatic treatments to select the best enzymatic treatment condition. Additionally, the extruder feed (corn bran) moisture was adjusted to three levels to select the best level of feed moisture content and use it for performing nine different extruding experiments. Concerning integrated extrusion‐enzymatic treatment, nine different extruding pretreatments were conducted on corn bran through selecting the optimum moisture level of extruding. Afterward, the product was milled and sieved to the optimum size for enzymatic treatment, and the nine pretreatments were combined with nine different enzymatic treatments. The product containing the highest soluble fiber was selected and used at various levels for functional cake production. Finally, some organoleptic and physicochemical properties (springiness, gumminess, hardness, cohesiveness, Bostwick number, density) of the produced product were analyzed.

## 1 INTRODUCTION

With the improvement of the standard of living, functional foods that can adjust the body function and prevent lifestyle diseases of civilization have attracted more attention in recent years. In this regard, due to their constructive effects for health, Dietary fiber (DF) plays an important role in human nutrition. For instance, it has been shown that consumption of DF decreased incidence of several diseases like heart disease, obesity, and cancers. Moreover, being indigestible in the small intestine, DF are responsible for fecal bulking and enhancing gut motility and they are utilized as fermentation substrates by the gut microflora (Jeddou et al., 2017; Yadav, Malik, Pathera, Islam, & Sharma, 2016).

Dietary fiber is generally derived from certain cereals, legumes, fruits, and vegetables and usually categorized as either water soluble or insoluble. Corn bran is an agro‐industrial derived by‐product, which arise during starch, flour, and high‐fructose corn syrup production and are associated with high DF content. Corn bran currently does not command a high price and traditionally uses for animal feed. Therefore, research is constantly underway to expand the use of these products and convert them into value‐added products. Destarched corn bran (DCB) contains cellulose (150–200 g/kg), hemicelluloses (400–500 g/kg), and lignin (80–130 g/kg), and since it is rich in arabinoxylan (70%), it is hence a good source for the production of xylooligosaccharides and other soluble dietary fibers (SDF) (Agger, Viksø‐Nielsen, & Meyer, 2010; Gáspár, Kálmán, & Réczey, 2007; Qiu et al., 2015; Yadav et al., 2016).

Physical, chemical, thermo‐chemical, biological, and enzymatic treatments can make the crud fiber highly digestible and hydrolyze hemicelluloses and remove lignin. These methods can be integrated together to enhance the efficiency of the digestion process. For instance, if the enzymatic method is chosen as the main digestion process, the alkaline pretreatment can delignify lignocelluloses (by disrupting the ester bonds cross‐linking lignin and xylan) efficiently and makes it more accessible for cellulolytic enzyme (Gáspár, Juhász, Szengyel, & Réczey, 2005; Singkhornart, Lee, & Ryu, 2013; Wang, Liu, Xie, Zhu, & Qin, 2018). On the other hand, extruding treatment is known as one of the effective and simple physical methods used for increasing the soluble fiber content of bran in which the materials (corn bran) are subjected to heating, mixing, and shearing, resulting in physical and chemical changes during the passage through the extruder barrel. High thermal dynamic efficiency, low operating cost, and ease of the scale up are some of the benefits of the extrusion method (Andersson, Andersson, Jonsäll, Andersson, & Fredriksson, 2017; Artz, Warren, & Villota, 1990; Yan, Ye, & Chen, 2015).

Limited research is devoted to production of SDF from corn fibers into forms for human consumption and most of the research has used nonintegrated treatments. Therefore, the research objectives were to use enzyme and extrusion treatments separately in order to increase the soluble fiber content of corn bran and investigate the effect of integrated treatments of enzyme and extrusion methods on enhancement of fiber digestion process. Moreover, the modified fiber was used for cake production to produce functional food, and then, a number of physicochemical and organoleptic properties of that product were investigated.

## 2 METHODS

### 2.1 Preparation of destarched corn bran

To prepare DCB, the same method of Singkhornart et al. (2013) was exactly performed.

### 2.2 Extrusion process

Extrusion treatment was performed using a pilot plant scale twin screw extruder (model D‐47055; OHG, Duisburg, Germany) with screw diameter (D) of 25 mm, which had a barrel length‐to‐screw diameter ratio (L/D) of 40:1, three heating zones, and die with one 3‐mm‐diameter hole. As Singkhornart et al. (2013) recommends, feed moisture content was adjusted to 30%, 40%, and 50%, and using one factor experiment, the temperature profile (140°C at all three heating zones) and rotation speed (300 rpm) of the helix were kept constant. After finding the optimum moisture level of the feed (from the standpoint of the highest soluble fiber content of the product), nine experiments were conducted by combining three temperature profiles (120, 140, and 160°C) and three replications of extrusion process (1, 2, and 3 times) at the fix helix rotation speed of 300 rpm. The extrusion products of these nine experiments were collected and analyzed in terms of soluble fiber content.

### 2.3 Alkali pretreatment

Destarched corn bran (100 g/L) was added to 1% (w/v) sodium hydroxide and autoclaved at 121°C for 1 hr. Then, the treated products were washed with water by using a sieve (mesh No. 100), and the remaining insoluble solids were dried at 45°C for 24 hr (Agger, Johansen, & Meyer, 2011).

### 2.4 Enzymatic hydrolysis

The remaining insoluble solid from alkaline pretreated DCB was dissolved (50 g/L) in 0.05 M sodium acetate buffer, and each of enzyme dose of 1%, 2.5%, and 5% was added to the mixture and shaken slowly (100 rpm) at 50°C for 48, 72, and 96 hr. The enzyme activity of celluclast 1.5 L, Viscozyme^®^ L and Pentopans 500 BG was 70 EGU (endoglucanase units) ml^−1^, 10 FBG (fungal β‐glucanase units) ml^−1^, and 270 FXU (fungal xylanase units) ml^−1^, respectively. The extrusion products of these nine experiments were collected and analyzed in terms of soluble fiber content (Agger et al., 2010).

### 2.5 Integrated extrusion‐enzymatic treatment of corn bran

In this stage of assay, the extrusion process was conducted as a pretreatment step before the enzymatic treatment to increase accessibility of cellulose for enzyme action (Singkhornart et al., 2013). In this regard, nine different treatments of extrusion process (combinations of three extrusion temperature and three replication of extruding) were conducted as pretreatment stages before nine different enzymatic processes (combinations of three enzyme doses and three periods of time). The final products of these 81 experiments were collected and analyzed in terms of soluble fiber content.

### 2.6 Production of corn fiber‐enriched cake

Using a household mixer (Model NS‐HM03; Newstar, China), whole eggs (56 g), sugar (75 g), and vanilla (0.45 g) were whipped completely at medium speed for 2 min. Afterward, at the same speed, 62.5 g of low‐fat milk was added and mixed until a thick cream was obtained. For three times, wheat flour (100 g) and 3.2 g of baking powder were sieved and then added to the cream gradually and mixed slightly. Different quantities of the wheat flour (5%, 10%, 15%, 20%, 25%, and 30%, W/W) were substituted by the modified corn bran for the highest SDF content from the previous sections, for corn fiber‐enriched cakes.

### 2.7 Determining the batter density and Bostwick number

Using a Bostwick consistometer at 20°C, the Bostwick number of the batter was determined. The distance moved by the batter (100 g) for 30 s was considered as Bostwick number, for which a negative correlation with batter consistency existed. In this respect, to determine the batter density, a glass tube with certain weight was first filled with batter, and the batter weight was determined. Then, the same glass tube was filled with distilled water, and the water volume was obtained. The batter density was obtained through the batter weight divided by the distilled water volume (Premi & Sharma, 2018).

### 2.8 Colorimetric analysis of the produced cake

Colorimetric analysis to measure effect of adding corn bran on the cake formulation was conducted by using a desktop hunterlab device (ColorFlex EZ, USA). Three parameters, L* (lightness), a* (redness), and b* (yellowness), were used to examine color changes. L* refers to the lightness of the samples, ranging from black = 0 to white = 100. A negative value for a* indicates green, while positive a* shows a red‐purple color. Moreover, positive and negative b* values show yellow and blue colors, respectively (Giménez, Gómez‐Estaca, Alemán, Gómez‐Guillén, & Montero, 2009).

### 2.9 Sensory analysis

For sensory evaluation of the produced cakes, appearance, flavor, taste, texture, and their overall acceptability were evaluated by 30 trained taste panelists on a five‐point scale: five for excellent, good for good, three for fair, two for poor, and one for terrible. This analysis was conducted using the Hedonic test described by Meilgaard, Carr, and Civille (2006).

### 2.10 Determination of the textural properties of the cakes

To determine the textural properties of the cakes, the crust was eliminated, and using a sharp knife, cubic pieces of the cakes (30 × 30 × 30 mm) were cut. Textural features of cake crumbs (30 × 30 × 30 mm) were measured using a TA‐XT2 texture analyzer (Stable Microsystems Ltd, Surrey, UK) provided with the software Texture Expert. An 80‐mm‐diameter metallic cylindrical probe was utilized in a Texture Profile Analysis (TPA) double compression test. The crumb was compacted to 25% of its initial height, at test speed of 0.25 mm/s and pretest speed of 5 mm/s, with a 10‐s delay between the first and second compression. From the TPA profile and according to descriptions of Majzoobi, Habibi, Hedayati, Ghiasi, and Farahnaky (2015), the peak force of the first compression cycle, cake hardness, cohesiveness, springiness, and gumminess were determined.

### 2.11 Other analytical methods

Total, insoluble, and SDF were determined according to the Association of Official Analytical Chemists (991.43, AOAC, 2000). Ash was determined by ignition in a muffle furnace (986.25, AOAC 2000); moisture was analyzed by drying for 3 hr at 130°C (935.29, AOAC, 2000); fat was determined by the Soxhlet procedure (905.02, AOAC, 2000), and protein amount was obtained using the Kjeldhal's method (991.20, AOAC, 2000). After determining the sample having the highest amount of soluble fiber, its pentose sugar content (as a major component of arabinoxylans) was determined by phloroglucinol method with xylose as a standard (Singkhornart et al., 2013). At last, the result was compared to that of raw corn bran.

### 2.12 Statistical analysis

Significant differences among all the treatments were analyzed using the one‐way ANOVA test (Duncan) at a significance level of 0.05, and all analyses were conducted in triplicate.

## 3 RESULTS AND DISCUSSION

### 3.1 Raw corn bran composition

The proximate analysis of corn bran indicated the following composition: protein 10.1 ± 0.2%, fat 4.2 ± 0.3%, ash 5.3 ± 0.2, total dietary fiber 62.4 ± 0.4, insoluble fiber 60.1 ± 0.1, soluble fiber 6.3 ± 0.3%, and starch 14.6%. These values correspond to those reported by Singkhornart et al. (2013).

### 3.2 Effect of extrusion process on SDF content of corn bran

Extrusion process induced disruption of the corn bran and allowed the cellulosic fiber to be easily separated (Artz et al., 1990). Table 1 summarizes the effects of feed moisture content, temperature profile of barrel and number of extruding replications on the SDF content of corn bran.

**Table 1:** The effects of extruding conditions on the soluble dietary fibers (SDF) content of corn bran

Treatment code	Feed moisture content (%), temperature profile of barrel (°C), rotation speed of helix (rpm), and number of extruding replications	SDF (%)
CB	—	3.2^e^ *
1 Ex CB	ECB 30%, 140°C, 300 rpm, 1 replication of extruding	9.2^bc^
2 Ex CB	ECB 40%, 140°C, 300 rpm, 1 replication of extruding	6.1^d^
3 Ex CB	ECB 50%, 140°C, 300 rpm, 1 replication of extruding	5.7^d^
4 Ex CB	ECB 30%, 120°C, 300 rpm, 1 replication of extruding	8.1^c^
5 Ex CB	ECB 30%, 140°C, 300 rpm, 1 replication of extruding	10.7^b^
6 Ex CB	ECB 30%, 160°C, 300 rpm, 1 replication of extruding	11.1^b^
7 Ex CB	ECB 30%, 120°C, 300 rpm, 2 replications of extruding	10.3^b^
8 Ex CB	ECB 30%, 140°C, 300 rpm, 2 replications of extruding	14.8^a^
9 Ex CB	ECB 30%, 160°C, 300 rpm, 2 replications of extruding	15.1^a^
10 Ex CB	ECB 30%, 120°C, 300 rpm, 3 replications of extruding	9.9^b^
11 Ex CB	ECB 30%, 140°C, 300 rpm, 3 replications of extruding	15.2^a^
12 Ex CB	ECB 30%, 160°C, 300 rpm, 3 replications of extruding	15.5^a^

*In each column, different superscript letters indicate significant differences (*p *<* *0.5).

As the results of Table 1 imply, in the fixed conditions of temperature, helix speed and number of extruding replications, the lower moisture content of the feed resulted in higher yield of the SDF content of corn bran. Therefore, the optimum moisture content of the feed equals to 30%, being similar to the findings of Singkhornart et al. (2013). Moreover, at the fixed moisture content of 30% and helix speed of 300, the highest SDF content of corn bran was obtained at 140°C through performing three replications of extruding (treatment number 11 Ex CB); however, since there is no significant difference (*p *<* *0.05) between SDF content of samples produced by treatments “11Ex CB” and “8 Ex CB,” and the number “8 Ex CB” treatment was chosen as the best condition of extruding condition (due to lower energy consumption).

### 3.3 Effect of enzyme hydrolysis on SDF content of DCB

The results of Table 2 indicate that after enzyme hydrolysis, among five different particle sizes of milled corn bran, the highest content of SDF was observed in the samples encoded “3, 4, and 5 En DCB,” and since there was no significant difference (*p *<* *0.05) between these three samples, the sample encoded “3 En DCB” was selected as the best sample in terms of mesh size and ease of sieving. To this stage, using one factor treatment, the best particle size (mesh number 60) of DCF was determined and used to perform the remaining nine enzymatic treatments. From the nine combinations of treatment times and enzymes doses, samples encoded “10, 11, 13, and 14 En DBC” had the highest content of SDF; however, since there was no significant difference (*p *<* *0.05) between these four selected samples, the sample encoded “10 En DCB” was selected as the best sample with high SDF yield.

**Table 2:** The effects of enzymatic treatment on the soluble dietary fibers (SDF) content of destarched corn bran (DCB)

Treatment code	Treatment time (hr), treatment temperature (°C), enzyme dose (%), and mesh number	SDF (%)
CB	—	3.2^c^ *
DCB	—	4.3^c^
1 En DCB	72 hr, 50°C, 2.5%, No. 20	14.2^b^
2 En DCB	72 hr, 50°C, 2.5%, No. 40	16.1^b^
3 En DCB	72 hr, 50°C, 2.5%, No. 60	21.2^a^
4 En DCB	72 hr, 50°C, 2.5%, No. 80	20.5^a^
5 En DCB	72 hr, 50°C, 2.5%, No. 100	22.6^a^
6 En DCB	48 hr, 50°C, 1%, No. 60	12.8^b^
7 En DCB	48 hr, 50°C, 2.5%, No. 60	16.5^b^
8 En DCB	48 hr, 50°C, 5%, No. 60	18.7^ab^
9 En DCB	72 hr, 50°C, 1%, No. 60	15.4^b^
10 En DCB	72 hr, 50°C, 2.5%, No. 60	21.2^a^
11 En DCB	72 hr, 50°C, 5%, No. 60	23.6^a^
12 En DCB	96 hr, 50°C, 1%, No. 60	16.1^b^
13 En DCB	96 hr, 50°C, 2.5%, No. 60	22.2^a^
14 En DCB	96 hr, 50°C, 5%, No. 60	23.8^a^

*In each column, different superscript letters indicate significant differences (*p *<* *0.5).

### 3.4 Effect of integrated extrusion‐enzymatic treatment on SDF content of DCB

Up to this stage, using extruding and enzyme digesting treatments, the highest SDF content of final product belonged to the samples encoded “8 Ex CD” and “10 En DCB.” In all extrusion pretreatments, the feed moisture content and rotation speed of helix were maintained fixed at 30% and 300 rpm, respectively. Moreover, all enzymatic treatments were performed at the fixed temperature and mesh number of 50°C and 60, respectively.

As Table 3 shows, in all of the 81 cases of study, integration of extruding and enzyme digesting treatments resulted in producing products with higher SDF content in contrast to extruding or enzyme digesting cures alone. The hypothesis is that extruder screw speed and barrel temperature may disturb the biomass structure, thereby increasing the accessibility of cellulose for enzyme action. Moreover, it can be inferred that integration of extruding and enzyme digesting treatments resulted in no change in TDF content. Among these 81 treatments, sample numbers 41, 42, 44, 45, 51, 53, 54, 68, 69, 71, 72, 77, 78, 80, and 81 had the highest SDF content; however, since there was no significant difference (*p *<* *0.05) between these top 15 samples, the sample encoded 41 Ex CB + En DECB was chosen as the best one (due to lower energy consumption, lower number of extruding replications, lower enzymatic treatment time, and lower enzyme dose added).

**Table 3:** The effects of integrated extrusion‐enzymatic treatment on the soluble dietary fibers (SDF) content of destarched corn bran (DCB)

Treatment code	Temperature profile of barrel (°C) and number of extruding replications	Enzyme dose (%)	SDF (%)	TDF (%)
CB	—	—	3.2^c^ *	62.5^a^
DCB	—	—	4.3^c^	62.1^a^
1 Ex CB + En DECB	120°C, 1 replication of extruding	48 hr, 1%	28.2^c^	61.9^a^
2 Ex CB + En DECB	120°C, 1 replication of extruding	48 hr, 2.5%	30.3^c^	62.3^a^
3 Ex CB + En DECB	120°C, 1 replication of extruding	48 hr, 5%	30.2^c^	62.4^a^
4 Ex CB + En DECB	120°C, 1 replication of extruding	72 hr, 1%	32.4^c^	62.2^a^
5 Ex CB + En DECB	120°C, 1 replication of extruding	72 hr, 2.5%	35.2^bc^	61.8^a^
6 Ex CB + En DECB	120°C, 1 replication of extruding	72 hr, 5%	36.6^bc^	62.3^a^
7 Ex CB + En DECB	120°C, 1 replication of extruding	96 hr, 1%	34^c^	61.9%
8 Ex CB + En DECB	120°C, 1 replication of extruding	96 hr, 2.5%	36.9^bc^	62.3^a^
9 Ex CB + En DECB	120°C, 1 replication of extruding	96 hr, 5%	38.8^b^	62.4^a^
10 Ex CB + En DECB	120°C, 2 replications of extruding	48 hr, 1%	34.6^c^	62.4^a^
11 Ex CB + En DECB	120°C, 2 replications of extruding	48 hr, 2.5%	36.^bc^	62.2^a^
12 Ex CB + En DECB	120°C, 2 replications of extruding	48 hr, 5%	36.2^bc^	62.0^a^
13 Ex CB + En DECB	120°C, 2 replications of extruding	72 hr, 1%	38.6^b^	62.5^a^
14 Ex CB + En DECB	120°C, 2 replications of extruding	72 hr, 2.5%	39.^b^	62.4^a^
15 Ex CB + En DECB	120°C, 2 replications of extruding	72 hr, 5%	40.2^b^	62.4^a^
16 Ex CB + En DECB	120°C, 2 replications of extruding	96 hr, 1%	40.8^b^	61.9^a^
17 Ex CB + En DECB	120°C, 2 replications of extruding	96 hr, 2.5%	41.2^b^	62.4^a^
18 Ex CB + En DECB	120°C, 2 replications of extruding	96 hr, 5%	41.8^b^	62.3^a^
19 Ex CB + En DECB	120°C, 3 replications of extruding	48 hr, 1%	39.6^b^	62.5^a^
20 Ex CB + En DECB	120°C, 3 replications of extruding	48 hr, 2.5%	40.1^b^	62.0^a^
21 Ex CB + En DECB	120°C, 3 replications of extruding	48 hr, 5%	42.2^b^	62.4^a^
22 Ex CB + En DECB	120°C, 3 replications of extruding	72 hr, 1%	40.8^b^	62.3^a^
23 Ex CB + En DECB	120°C, 3 replications of extruding	72 hr, 2.5%	43.5^b^	62.1^a^
24 Ex CB + En DECB	120°C, 3 replications of extruding	72 hr, 5%	43.7^b^	62.2^a^
25 Ex CB + En DECB	120°C, 3 replications of extruding	96 hr, 1%	41.4^b^	62.4^a^
26 Ex CB + En DECB	120°C, 3 replications of extruding	96 hr, 2.5%	42.2^b^	61.9^a^
27 Ex CB + En DECB	120°C, 3 replications of extruding	96 hr, 5%	43.6^b^	62.3^a^
28 Ex CB + En DECB	140°C, 1 replication of extruding	48 hr, 1%	33.1^c^	61.9^a^
29 Ex CB + En DECB	140°C, 1 replication of extruding	48 hr, 2.5%	37.2^bc^	62.1^a^
30 Ex CB + En DECB	140°C, 1 replication of extruding	48 hr, 5%	38.8^b^	62.2^a^
31 Ex CB + En DECB	140°C, 1 replication of extruding	72 hr, 1%	35.6^bc^	62.4^a^
32 Ex CB + En DECB	140°C, 1 replication of extruding	72 hr, 2.5%	39.1^b^	62.3^a^
33 Ex CB + En DECB	140°C, 1 replication of extruding	72 hr, 5%	40.2^b^	61.9^a^
34 Ex CB + En DECB	140°C, 1 replication of extruding	96 hr, 1%	36.6^bc^	62.3^a^
35 Ex CB + En DECB	140°C, 1 replication of extruding	96 hr, 2.5%	40.1^b^	62.3^a^
36 Ex CB + En DECB	140°C, 1 replication of extruding	96 hr, 5%	41.6^b^	62.0^a^
37 Ex CB + En DECB	140°C, 2 replications of extruding	48 hr, 1%	39.8^b^	61.9^a^
38 Ex CB + En DECB	140°C, 2 replications of extruding	48 hr, 2.5%	42.2^b^	62.4^a^
39 Ex CB + En DECB	140°C, 2 replications of extruding	48 hr, 5%	44.8^ab^	62.2^a^
40 Ex CB + En DECB	140°C, 2 replications of extruding	72 hr, 1%	42.6^b^	62.1^a^
41 Ex CB + En DECB	140°C, 2 replications of extruding	72 hr, 2.5%	48.3^a^	62.4^a^
42 Ex CB + En DECB	140°C, 2 replications of extruding	72 hr, 5%	51.8^a^	62.2^a^
43 Ex CB + En DECB	140°C, 2 replications of extruding	96 hr, 1%	42.8^b^	62.3^a^
44 Ex CB + En DECB	140°C, 2 replications of extruding	96 hr, 2.5%	49.6^a^	61.9^a^
45 Ex CB + En DECB	140°C, 2 replications of extruding	96 hr, 5%	52.8^a^	62.4^a^
46 Ex CB + En DECB	140°C, 3 replications of extruding	48 hr, 1%	40.4^b^	62.0^a^
47 Ex CB + En DECB	140°C, 3 replications of extruding	48 hr, 2.5%	43.2^b^	62.2^a^
48 Ex CB + En DECB	140°C, 3 replications of extruding	48 hr, 5%	45.6^ab^	62.2^a^
49 Ex CB + En DECB	140°C, 3 replications of extruding	72 hr, 1%	43.6^b^	62.1^a^
50 Ex CB + En DECB	140°C, 3 replications of extruding	72 hr, 2.5%	47.9^ab^	61.9^a^
51 Ex CB + En DECB	140°C, 3 replications of extruding	72 hr, 5%	52.2^a^	62.3^a^
52 Ex CB + En DECB	140°C, 3 replications of extruding	96 hr, 1%	43.6^b^	62.3^a^
53 Ex CB + En DECB	140°C, 3 replications of extruding	96 hr, 2.5%	48.9^a^	62.2^a^
54 Ex CB + En DECB	140°C, 3 replications of extruding	96 hr, 5%	53.2^a^	62.0^a^
55 Ex CB + En DECB	160°C, 1 replication of extruding	48 hr, 1%	36.8^a^	62.2^a^
56 Ex CB + En DECB	160°C, 1 replication of extruding	48 hr, 2.5%	39.2^b^	61.9^a^
57 Ex CB + En DECB	160°C, 1 replication of extruding	48 hr, 5%	40.8^b^	62.3^a^
58 Ex CB + En DECB	160°C, 1 replication of extruding	72 hr, 1%	37.6^bc^	62.4^a^
59 Ex CB + En DECB	160°C, 1 replication of extruding	72 hr, 2.5%	40.1^b^	62.3^a^
60 Ex CB + En DECB	160°C, 1 replication of extruding	72 hr, 5%	41.2^b^	62.3^a^
61 Ex CB + En DECB	160°C, 1 replication of extruding	96 hr, 1%	37.8^bc^	62.1^a^
62 Ex CB + En DECB	160°C, 1 replication of extruding	96 hr, 2.5%	39.9^b^	61.9^a^
63 Ex CB + En DECB	160°C, 1 replication of extruding	96 hr, 5%	41.7^b^	62.2^a^
64 Ex CB + En DECB	160°C, 2 replications of extruding	48 hr, 1%	40.4^b^	62.0^a^
65 Ex CB + En DECB	160°C, 2 replications of extruding	48 hr, 2.5%	42.5^b^	62.2^a^
66 Ex CB + En DECB	160°C, 2 replications of extruding	48 hr, 5%	45.6^ab^	62.4^a^
67 Ex CB + En DECB	160°C, 2 replications of extruding	72 hr, 1%	43.3^b^	61.9^a^
68 Ex CB + En DECB	160°C, 2 replications of extruding	72 hr, 2.5%	49.2^a^	62.1^a^
69 Ex CB + En DECB	160°C, 2 replications of extruding	72 hr, 5%	52.4^a^	62.2^a^
70 Ex CB + En DECB	160°C, 2 replications of extruding	96 hr, 1%	43.1^b^	62.2^a^
71 Ex CB + En DECB	160°C, 2 replications of extruding	96 hr, 2.5%	49.3^a^	62.1^a^
72 Ex CB + En DECB	160°C, 2 replications of extruding	96 hr, 5%	53.4^a^	62.1^a^
73 Ex CB + En DECB	160°C, 3 replications of extruding	48 hr, 1%	41.1^b^	62.4^a^
74 Ex CB + En DECB	160°C, 3 replications of extruding	48 hr, 2.5%	42.9^b^	61.9^a^
75 Ex CB + En DECB	160°C, 3 replications of extruding	48 hr, 5%	46.3^ab^	62.3^a^
76 Ex CB + En DECB	160°C, 3 replications of extruding	72 hr, 1%	43.9^b^	62.2^a^
77 Ex CB + En DECB	160°C, 3 replications of extruding	72 hr, 2.5%	48.1^a^	62.0^a^
78 Ex CB + En DECB	160°C, 3 replications of extruding	72 hr, 5%	52.7^a^	62.1^a^
79 Ex CB + En DECB	160°C, 3 replications of extruding	96 hr, 1%	43.9^b^	62.3^a^
80 Ex CB + En DECB	160°C, 3 replications of extruding	96 hr, 2.5%	49.6^a^	62.4^a^
81 Ex CB + En DECB	160°C, 3 replications of extruding	96 hr, 5%	53.6^a^	62.1^a^

*In each column, different superscript letters indicate significant differences (*p *<* *0.5).

### 3.5 Corn fiber effect on batter properties

**Figure 1:**
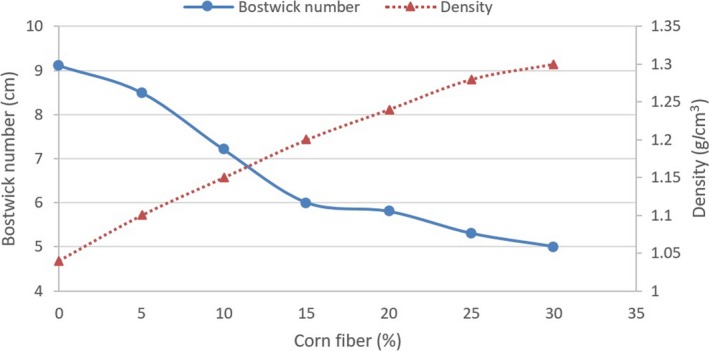
Bostwick number and density of the cake batter containing different levels of corn fiber

Based on the findings (Figure 1), the Bostwick number decreased from 9.11 to 5.00 cm with increasing the corn bran content in cake formulation. Bostwick number is negatively correlated with consistency; therefore, by adding the corn fiber, the batter consistency increased. The increase in the batter consistency was associated with the corn fiber high water absorption. Moreover, consistent with this study, Rosell, Santos, and Collar (2009) stated the increase of cake batter density by adding insoluble fibers. Figure 1 also showed that batter density increased from 1.04 to 1.30 g/cm^3^ with increase in the corn fiber level, indicating that less air bubbles were probably entrapped in the batter.

### 3.6 Effects of corn fiber on physical properties of the cakes

Table 4 presents the effect of fiber on the textural properties of the cake. By measuring hardness (firmness), the maximum force required for disintegration of the cake structure is obtained. Gumminess represents the required energy for breakdown of the cake to a state ready for swallowing. By cohesiveness, the internal resistance of the cake structure was measured and by springiness (elasticity), the ease of returning to the original size of a piece of cake after being slightly pressed was dignified (Majzoobi et al. (2015). Based on the findings, increasing the percentage of the corn fiber caused reduction in the cohesiveness and springiness of the cakes. Moreover, as it was also stated by Grigelmo‐Miguel, Carreras‐Boladeras, and Martín‐Belloso (1999), increasing the corn fiber content was conducted to increase gumminess factor, but resulted in no significant change (*p *<* *0.5) in hardness when corn bran is added up to 20% of cake formulation.

**Table 4:** Effect of different contents of corn bran on textural properties of the cake

Corn fiber (%)	Springiness (mm)	Gumminess (kg)	Hardness (kg)	Cohesiveness
0	0.887 ± 0.003^a^ *	0.416 ± 0.002^c^	5.62 ± 0.09^b^	0.857 ± 0.004^a^
5	0.862 ± 0.002^a^	0.425 ± 0.002^c^	5.94 ± 0.0.7^ab^	0.813 ± 0.003^a^
10	0.836 ± 0.002^ab^	0.437 ± 0.001^bc^	6.03 ± 0.08^ab^	0.763 ± 0.002^b^
15	0.814 ± 0.003^b^	0.467 ± 0.002^b^	6.09 ± 0.10^ab^	0.726 ± 0.003^bc^
20	0.792 ± 0.001^b^	0.481 ± 0.003^b^	6.17 ± 0.12^ab^	0.687 ± 0.002^c^
25	0.788 ± 0.002^b^	0.496 ± 0.003^ab^	6.46 ± 0.11^a^	0.636 ± 0.003^d^
30	0.781 ± 0.002^b^	0.513 ± 0.002^a^	6.57 ± 0.13^a^	0.587 ± 0.001^e^

*In each column, different superscript letters indicate significant differences (*p *<* *0.5).

### 3.7 Effect of corn fiber concentration on color characteristics of cakes

Since crust color is influenced by Maillard and caramelization reactions, adding corn bran to the cake formulation resulted in lower L‐values (darker crust color), higher b‐values (more yellowish), and lower a‐values (less reddish) of the crust (Table 5). Furthermore, in the inner parts of the cake (crump) where the temperature was lower than that of the crust, Maillard and caramelization reactions did not provided and therefore the color was highly dependent on raw materials. It was observed that increasing the corn bran percentage more than 5% of cake formulation caused the L‐ and a‐values and the b‐value of the crump to be significantly (*p *<* *0.5) reduced and increased, respectively.

**Table 5:** Effect of different contents of corn bran on cake color

Corn fiber (%)	Crust			Crump		
	L‐value	a‐Value	b‐Values	L‐value	a‐Value	b‐Values
0	38.4 ± 0.8^a^ *	9.1 ± 0.4^a^	24.3 ± 0.6^b^	87.4 ± 1.2^a^	−8.7 ± 0.2^a^	36.8 ± 0.8^e^
5	37.2 ± 0.5^a^	8.3 ± 0.6^b^	25.1 ± 0.7^b^	83.3 ± 0.9^a^	−9.3 ± 0.3^a^	37.2 ± 0.7^e^
10	32.8 ± 0.7^b^	7.9 ± 0.5^b^	27.1 ± 0.8^ab^	76.8 ± 1.0^b^	−10.8 ± 0.3^b^	38.7 ± 0.7^d^
15	31.3 ± 0.9^b^	5.9 ± 0.6^c^	27.7 ± 1.0^ab^	70.5 ± 0.7^c^	−11.2 ± 0.5^b^	39.2 ± 0.9^d^
20	30.1 ± 0.8^bc^	4.4 ± 0.3^d^	29.9 ± 0.6^a^	68.6 ± 0.8^c^	−12.5 ± 0.4^bc^	39.9 ± 0.8^c^
25	28.8 ± 1.0^c^	2.3 ± 0.3^e^	31.2 ± 1.1^a^	61.8 ± 0.8^d^	−13.9 ± 0.7^c^	40.4 ± 0.6^b^
30	27.1 ± 0.6^c^	1.1 ± 0.2^f^	32.7 ± 0.9^a^	57.9 ± 0.4^d^	−14.6 ± 0.6^c^	41.4 ± 1.1^a^

*In each column, different superscript letters indicate significant differences (*p *<* *0.5).

### 3.8 Sensory evaluation

As results in Table 6 demonstrate, the best taste and texture properties of the product were achieved when 10% and 15% of corn bran were added to the cake formulation, while adding 30% corn bran led to inferior taste and texture of the cake. The color and odor intensity of the cake samples were the causes of appearance and flavor scores, respectively. Regarding appearance analysis, the higher corn bran percentage in the cake formulation, the lower scores were assigned, but still in an acceptable range (up to adding 10% of corn bran). Adding corn bran, for flavor analysis, did not have any significant (*p *<* *0.05) effect on the given scores. Moreover, according to the results of the overall acceptability tests, the presence of 5% of corn bran led to products with higher appreciation by the panelists. Furthermore, the cakes containing 30% corn bran overall involved the lowest acceptability compared to the other samples.

**Table 6:** Effect of different contents of corn fiber on sensory properties of cake

Corn bran (%)	Taste	Texture	Crump appearance	Crust appearance	Flavor	Overall acceptability
0	3.7 ± 0.1^c^ *	3.7 ± 0.1^bc^	4.6 ± 0.3^a^	4.6 ± 0.2^a^	4.3 ± 0.2^ab^	4.3 ± 0.1^ab^
5	4.2 ± 0.1^b^	3.9 ± 0.1^b^	4.7 ± 0.1^a^	4.5 ± 0.1^a^	4.4 ± 0.1^a^	4.5 ± 0.3^a^
10	4.7 ± 0.2^a^	4.1 ± 0.2^ab^	4.6 ± 0.1^a^	4.4 ± 0.1^ab^	4.2 ± 0.1^b^	4.2 ± 0.2^ab^
15	4.4 ± 0.3^ab^	4.5 ± 0.1^a^	4.3 ± 0.2^ab^	4.1 ± 0.2^b^	4.3 ± 0.1^ab^	3.9 ± 0.1^b^
20	4.2 ± 0.1^b^	4.0 ± 0.3^b^	4.0 ± 0.1^b^	4.0 ± 0.1^b^	4.3 ± 0.2^ab^	4.0 ± 0.4^b^
25	3.4 ± 0.1 ^cd^	4.0 ± 0.1^b^	4.1 ± 0.2^b^	3.8 ± 0.1^bc^	4.5 ± 0.1^a^	3.7 ± 0.1^c^
30	2.6 ± 0.1^d^	3.1 ± 0.1^c^	3.8 ± 0.1^c^	3.6 ± 0.1^c^	4.4 ± 0.1^a^	3.3 ± 0.1^d^

*In each column, different superscript letters indicate significant differences (*p *<* *0.5).

## 4 CONCLUSION

The increase in fiber content of high‐consumption food can compensate for shortage of the fiber in the diet. In this regard, the increase of soluble fiber content of corn bran and its application in cake formulation was achieved using extruding and enzymatic treatments. Extruding and enzymatic treatments successfully increased the SDF content of corn bran, and when these methods were used with each other, a synergistic effect was observed, and higher amounts of SDF content were measured in the product. Moreover, inclusion of the modified corn bran in the cake formulation resulted in producing a functional cake with lower cohesiveness and springiness higher gumminess, darkness, and more desirable sensory properties. Overall, based on the analysis of this study, adding 5% to 10% of modified corn bran to cake formulation can result in producing a functional cake with superior sensory and textural properties than cakes lacking modified corn bran in their formulation.

## CONFLICT OF INTEREST

None declared.

## ETHICAL STATEMENT

There is no conflict of interest, and all authors are in agreement to submit the present manuscript to the journal of “Food Science & Nutrition”. Furthermore, the manuscript does not contain experiments using animals or human.
